# Elevated Cholinesterase Activity and the Metabolic Syndrome—Dissecting Fatty Liver, Insulin Resistance and Dysglycaemia

**DOI:** 10.1111/liv.70046

**Published:** 2025-04-17

**Authors:** Martin Heni, Julia Hummel, Louise Fritsche, Robert Wagner, Lasse Relker, Jürgen Machann, Fritz Schick, Andreas L. Birkenfeld, Erwin Schleicher, Alfred Königsrainer, Hans‐Ulrich Häring, Norbert Stefan, Andreas Fritsche, Andreas Peter

**Affiliations:** ^1^ Division of Endocrinology and Diabetology, Department of Internal Medicine I University of Ulm Ulm Germany; ^2^ Department for Diagnostic Laboratory Medicine, Institute for Clinical Chemistry and Pathobiochemistry Eberhard Karls University Tübingen Tübingen Germany; ^3^ Institute for Diabetes Research and Metabolic Diseases of the Helmholtz Center Munich University of Tübingen Tübingen Germany; ^4^ German Center for Diabetes Research (DZD) Neuherberg Germany; ^5^ Division of Diabetology, Endocrinology and Nephrology, Department of Internal Medicine IV Eberhard Karls University Tübingen Tübingen Germany; ^6^ Division of Endocrinology and Diabetology, Medical Faculty Heinrich Heine University Düsseldorf Germany; ^7^ Institute for Clinical Diabetology, German Diabetes Center, Leibniz Institute for Diabetes Research Heinrich Heine University Düsseldorf Germany; ^8^ Department of Radiology, Section on Experimental Radiology Eberhard Karls University Tübingen Tübingen Germany; ^9^ Department of General, Visceral and Transplant Surgery Eberhard Karls University Tübingen Tübingen Germany

**Keywords:** cholinesterase, insulin sensitivity, liver fat, metabolic syndrome

## Abstract

**Background and Aims:**

While low plasma butyrylcholinesterase (BChE) is a well‐established marker of reduced liver synthesis capacity, the clinical significance of elevated BChE is unclear. In small studies, high BChE has long been suspected in hepatic steatosis and metabolic syndrome. We aimed to clarify the relation between BChE, liver fat and glucose metabolism in deeply phenotyped cohorts.

**Methods:**

Plasma BChE activity was measured in 844 humans (554 women) of the cross‐sectional Tübingen Diabetes Family Study, with a wide BMI range (17.7–55.1 kg/m^2^). It was furthermore measured before and after two independent lifestyle intervention studies in 215 and 116 participants. Liver fat was quantified with ^1^H‐MR‐spectroscopy, and metabolism was assessed by oral glucose tolerance tests.

**Results:**

BChE was positively associated with liver fat, independent of sex, age and BMI. BChE was higher in participants with metabolic syndrome. BChE was positively associated with fasting and 2‐h glycaemia, independent of sex, age and BMI. BChE was negatively associated with insulin sensitivity, independent of sex, age, BMI and liver fat. The reduction of liver fat and improvement in insulin sensitivity during lifestyle interventions are associated with the reduction in BChE, independent of body weight loss.

**Conclusions:**

Higher plasma BChE activity is linked to liver fat accumulation, as well as impaired glucose tolerance and insulin resistance, independent of liver fat. This suggests that BChE could be a marker for processes in hepatocytes that contribute to impaired glucose metabolism. Further investigations are needed to clarify the mechanistic contribution and potential diagnostic value of elevated BChE in hepatic steatosis and metabolic diseases.

**Summary**

- Elevated butyrylcholinesterase (BChE) is linked to liver fat, insulin resistance and glucose intolerance, independent of each other.
- Reducing (BChE) through lifestyle interventions improves liver fat and insulin sensitivity, particularly in lean, young individuals at risk of fatty liver disease.

**Abbreviations**
AChE: acetylcholinesterase
AUC: area under the curve
BChE: butyrylcholinesterase
BIA: bioelectrical impedance analysis
BMI: body mass index
cDNA: complementary DNA
CI: confidence interval
HbA1c: haemoglobin A1c
HDL‐cholesterol: high‐density lipoprotein cholesterol
IQR: interquartile range
LDL‐cholesterol: low‐density lipoprotein cholesterol
MRI: magnetic resonance imaging
mRNA: messenger ribonucleic acid
MRS: magnetic resonance spectroscopy
NUPREDM: Nutritional Prevention of Diabetes Mellitus Type 2
OGTT: oral glucose tolerance test
PCR: polymerase chain reaction
RNA: ribonucleic acid
SD: standard deviation
TULIP: Tübingen Lifestyle Intervention Program

## 1 Introduction

There are two closely homologous main types of the enzyme family of cholinesterases—acetylcholinesterase (AChE) and butyrylcholinesterase (BChE, also known as pseudo‐cholinesterase) [1]. AChE has a specific and well‐defined function at the synapse and neuromuscular junction, where it rapidly hydrolyses acetylcholine to terminate the transmission of nerve impulses [1, 2].

BChE function is, however, less clear. It has the ability to compensate for AChE but hydrolyses several other substrates and functions as a detoxifier, for example for nerve agents and pesticides [1]. BChE synthesis in the liver is a major source of circulating BChE [2]. In clinical practice, the determination of plasma BChE activity is mainly used to assess liver synthesis performance. Low circulating levels of BChE are present in liver failure, while the importance of elevated levels is still not fully understood [2].

Excess fat accumulation in the liver is an important contributor to the development and progression of metabolic syndrome and type 2 diabetes. Previous small studies suggested elevated levels of BChE in patients with fatty liver [3, 4], obesity [3, 5, 6], and metabolic syndrome [6, 7]. For the link to glycaemia and type 2 diabetes, reports are inconsistent, with some reporting higher [8, 9], while others report lower BChE activity [7]. Thus, the precise role of BChE in systemic glucose metabolism and metabolic diseases is still unclear.

Therefore, we examined the relationships of BChE activity with glucose metabolism and metabolic syndrome in cross‐sectional and two longitudinal studies. Additionally, we determined the relationship between BChE activity and precisely quantified liver fat content.

## 2 Methods

### 2.1 Cross‐Sectional Study

For cross‐sectional analyses, 844 participants of the ongoing Tübingen Diabetes Family Study [10] without severe diseases (including liver diseases) were included. They underwent precise metabolic phenotyping, including a whole‐body MRI [10] and liver ^1^H‐MRS [11] as well as a 2‐h 75 g glucose oral glucose tolerance test (OGTT). The presence of the metabolic syndrome was defined according to the joint interim statement of the International Diabetes Federation Task Force on Epidemiology and Prevention; National Heart, Lung, and Blood Institute; American Heart Association; World Heart Federation; International Atherosclerosis Society and International Association for the Study of Obesity [12]. All participants provided informed written consent, and the Ethics Committee of the University of Tübingen approved the study protocol. Participant characteristics are presented in Table 1.

**TABLE 1:** Participant characteristics—Cross‐sectional cohort.

	*N*	Median	IQR
Sex			
Female	554		
Male	290		
Age (years)	844	43	(33, 54)
BMI (kg/m^2^)	844	29.2	(25.6, 32.9)
Waist to hip ratio	843	0.9	(0.8, 1.0)
Body fat, BIA‐derived (%)	823	34.4	(25.6, 44.5)
Total adipose tissue, MR‐derived (L)	836	33.7	(24.9, 43.9)
Intrahepatic fat, MRS‐derived (%)	844	3.2	(1.4, 9.0)
HbA1c (%)	819	5.6	(5.3, 5.8)
Fasting glucose (mmol/L)	844	5.2	(4.9, 5.7)
2‐h glucose (mmol/L)	844	6.6	(5.6, 7.9)
Area under the glucose curve, 0–120 min (mmol/L)	843	15.4	(13.4, 18.1)
Matsuda insulin sensitivity index (OGTT‐derived)	840	11.9	(7.3, 18.2)
AUC C‐peptide_(0‐30min)_/AUC glucose_(0‐30min)_	839	165	(127, 213)
Butyrylcholinesterase (kU/L)	844	8.8	(7.3, 10.5)
Aspartate aminotransferase (U/L)	837	22	(17, 27)
Alanine aminotransferase (U/L)	841	22	(16, 32)
Gamma‐glutamyltransferase (U/L)	843	18	(12, 30)
Albumin (g/dL)	719	4.3	(4.1, 4.5)
Free fatty acids (μmol/L)	839	610	(485, 764)
Triglycerides (mg/dL)	843	100	(72, 136)
Cholesterol (mg/dL)	843	188	(164, 214)
LDL‐cholesterol (mg/dL)	842	115	(95, 138)
HDL‐cholesterol (mg/dL)	842	51	(44, 60)

Abbreviations: AUC: area under the curve; BIA: bioelectrical impedance analysis; BMI: body mass index; HbA1c: haemoglobin A1c; HDL‐cholesterol: high‐density lipoprotein cholesterol; LDL‐cholesterol: low‐density lipoprotein cholesterol; MR: magnetic resonance; MRS: magnetic resonance spectroscopy; OGTT: oral glucose tolerance test.

### 2.2 Lifestyle Intervention Studies

For the longitudinal analyses during lifestyle intervention, data from two independent trials in participants at increased risk for type 2 diabetes were analysed.

The TULIP (Tübingen Lifestyle Intervention Program) trial combined exercise and diet modification [13]. We analysed data from 215 persons with available plasma before and after 9 months of the intervention. These subjects achieved a median weight loss of 2.3 kg (IQR 4.2) and a median absolute reduction of liver fat content of 0.6% (IQR 2.2). For other participant characteristics, see Table S1.

The NUPREDM (Nutritional Prevention of Diabetes Mellitus Type 2) study investigated the effect of modification in red meat and fiber intake, compared to a control intervention, over 6 months [14]. We analysed data of 116 participants with available plasma, of whom complete data were available. These persons achieved a median weight loss of 2.3 kg (IQR 5.6) and a median absolute reduction of liver fat content of 0.8% (IQR 2.3). For further participant characteristics, see Table S1. As body weight loss and reduction of liver fat content were comparable between the three investigated diets in this trial [14], we analysed pooled data and did not stratify for the three diets.

### 2.3 Measurements

All participants underwent a 75 g OGTT after an overnight fast with blood sampling at 0, 30, 60, 90 and 120 min. These samples were stored at −80°C.

BChE activity was measured photometrically (Butyrylthiocholin 5‐Thio‐2‐nitrobenzoat) in lithium‐heparin plasma that was stored at –80°C on an ADVIA XPT clinical chemistry analyser (Siemens Healthineers, Eschborn, Germany). Albumin, triglycerides, total cholesterol, HDL‐cholesterol, LDL‐cholesterol and further clinical chemistry were also measured on the latter instrument.

Serum insulin and C‐peptide concentrations were measured on the ADVIA Centaur XPT immunoassay analyser (Siemens Healthineers, Eschborn, Germany) and glycated haemoglobin (HbA1c) was measured using the Tosoh A1c analyser HLC‐723G8 (Tosoh Bioscience GmbH, Griesheim, Germany). All of these measurements have been performed in the accredited diagnostic laboratory of the University Hospital Tübingen, Germany.

Insulin sensitivity was estimated from insulin and glucose during the OGTT as proposed by Matsuda and DeFronzo [15]. Oral glucose‐induced insulin secretion was estimated using the areas under the C‐peptide and glucose curves during the first 30 min of the OGTT (AUC C‐peptid_0‐30_/AUC glucose_0‐30_) and analysed after adjustment for insulin sensitivity [16].

### 2.4 Determination of Liver Fat Content In Vivo

Liver fat content was measured by ^1^H‐MR‐spectroscopy as described previously [11].

### 2.5 Determination of Liver Fat Content In Vitro

A total of 238 individuals who underwent liver surgery (e.g., for the resection of solitary hepatic lesions) were included in the present study (Department of General, Visceral and Transplant Surgery at the University of Tübingen). Patient characteristics are shown in Table S2. Patients were fasted overnight before the collection of the liver biopsies and corresponding blood samples. Liver samples were taken from normal, nondiseased tissue as determined by the pathologist during surgery. Samples were immediately frozen in liquid nitrogen and stored at −80°C until analysis. All patients tested negative for viral hepatitis and had no liver cirrhosis. Informed written consent was obtained from all participants, and the local medical ethics committee approved the protocol according to the Helsinki Declaration.

### 2.6 Determination of Liver Tissue Triglyceride Content

Liver tissue samples were homogenised in phosphate‐buffered saline containing 1% Triton X‐100 with a TissueLyser (Qiagen, Hilden, Germany). To determine liver fat content, triglyceride concentrations in the homogenates were quantified using an ADVIA XPT clinical chemistry analyser (Siemens Healthineers, Eschborn, Germany), and the results were calculated as mg/100 mg tissue weight (%) [17].

### 2.7 Real‐Time PCR

Frozen tissue was homogenised in a TissueLyser (Qiagen, Venlo, The Netherlands) and BChE RNA was extracted with the RNeasy Tissue Kit (Qiagen, Venlo, The Netherlands) according to the manufacturer's instructions. Total RNA treated with RNase‐free DNase I was transcribed into cDNA by using a first‐strand cDNA kit, and PCRs were performed in duplicates on a LightCycler480 (Roche Diagnostics, Mannheim, Germany). The human primer sequences that were used are shown in Table S3. Data are presented relative to the expression of the housekeeping gene *RPS13* using the ΔΔCt method.

### 2.8 Statistical Methods

Statistical analyses were performed using R, version 4.2.2. Data were log‐transformed prior to statistical analyses. Groups were compared by two‐tailed unpaired t‐tests. Associations were assessed by multivariable linear regression models. Standardised betas were calculated using standardised variables (scaled by subtracting the mean and dividing by the SD). *p* < 0.05 was considered to indicate statistical significance.

## 3 Results

### 3.1 Cross‐Sectional Analyses

**FIGURE 1:**
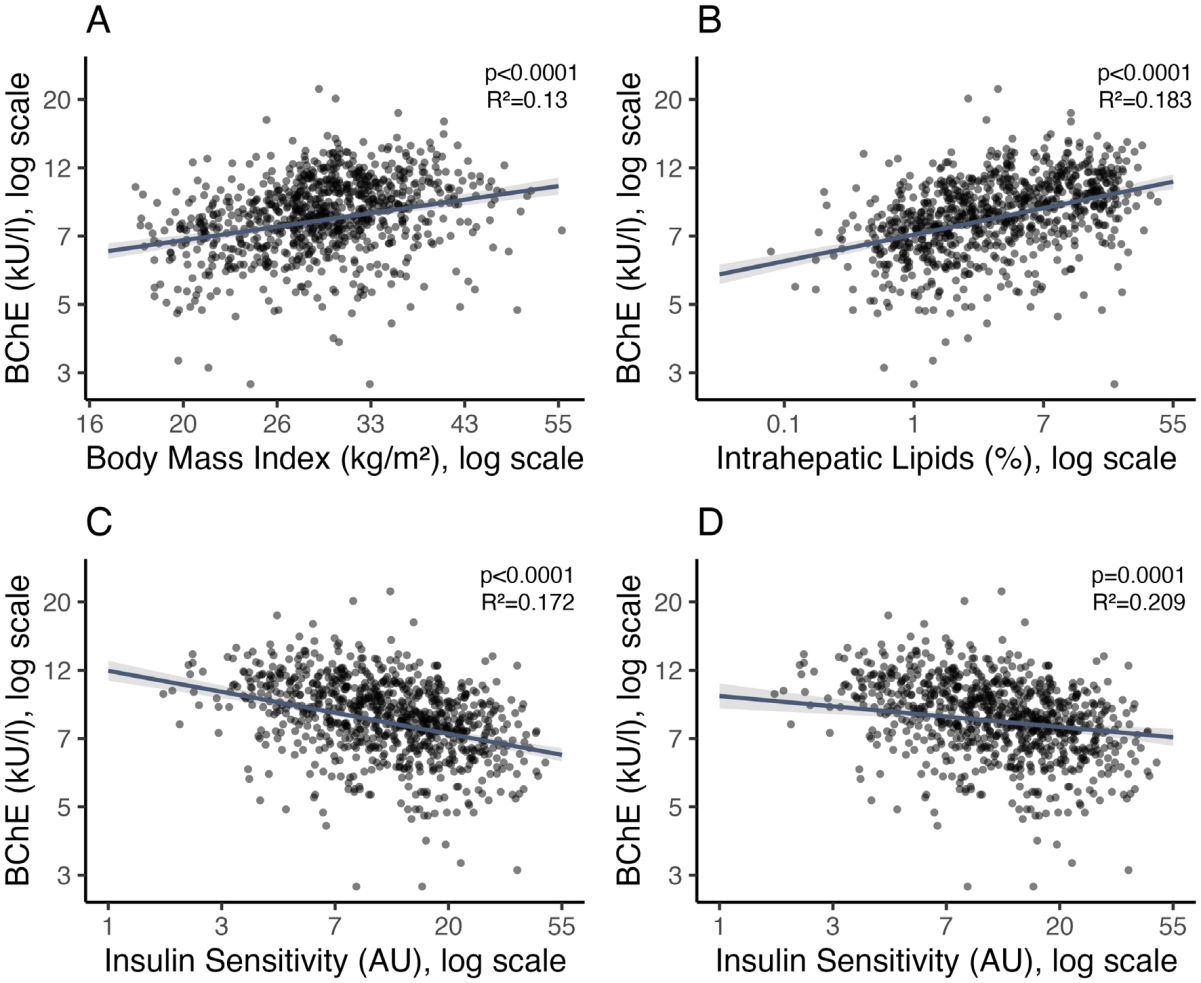
Associations of butyrylcholinesterase activity with BMI, liver fat content and insulin sensitivity: Cross‐sectional results. Butyrylcholinesterase (BChE) was positively associated with BMI, adjusted for sex and age (A). There was a positive correlation between BChE and liver fat content, adjusted for sex and age (B). BChE was negatively associated with insulin sensitivity, adjusted for sex and age (C). This association remained significant after additional adjustment for BMI and liver fat content (D). Plotted are marginal effects from multivariable regression models, including regression lines and 95% CI (*N* = 844). Data were log‐transformed prior to statistical analyses. Axes indicate nontransformed data. *p* values and *R* ^2^ are from multivariable linear regression analyses. BChE—butyrylcholinesterase, BMI—body mass index, CI—confidence interval.

Plasma BChE activity was measured in 844 subjects with wide ranges of age (18–74 years) and BMI (17.7–55.1 kg/m^2^; for further participant characteristics, see Table 1). BChE activity was higher in men than in women (women: 8.4 kU/L [IQR 2.9], men: 9.7 kU/L [IQR 3.5], *p* < 0.0001) and higher in older persons (*p* < 0.0001, *R* ^2^ = 0.03). It was positively associated with BMI, independent of age and sex (*p* < 0.0001, Figure 1A).

There was a positive association between BChE and liver fat content, independent of age and sex (*p* < 0.0001, Figure 1B). This association remained significant after additional adjustments for BMI (*p* < 0.0001, *R* ^2^ = 0.192) and insulin sensitivity (*p* < 0.0001, *R* ^2^ = 0.209).

BChE was negatively associated with insulin sensitivity, independent of sex and age (*p* < 0.0001, Figure 1C), as well as after additional adjustment for BMI and liver fat content (*p* = 0.0001, Figure 1D, Table S4). The association between BChE activity and insulin secretion (assessed as AUC C‐peptid_0‐30_/AUC glucose_0‐30_) was fully explained by differences in insulin sensitivity and did not remain significant after adjustment for insulin sensitivity (*p* = 0.8, for further adjustments see Table S4).

Associations of BChE activity with both fasting and postload glycaemia (all *p* < 0.0001, Table S4) were observed that remained significant after additional adjustments for BMI and liver fat content (all *p* < 0.0001, Table S4).

BChE was higher in subjects with the metabolic syndrome (*p* < 0.0001, 8.0 kU/L [IQR 2.6] vs. 9.9 kU/L [IQR 3.1], Figure S1), which remained significant after adjustment for sex and age (*p* < 0.0001).

Furthermore, there were significant associations of BChE activity with parameters of lipid metabolism, with positive associations with triglycerides, total and LDL‐cholesterol, and negative association with HDL‐cholesterol (Table S4).

As an estimate of general liver synthesis capacity, we additionally analysed plasma albumin concentrations. BChE activity and albumin levels were positively correlated (*p* < 0.0001, *R* ^2^ = 0.05). However, there were no significant correlations of plasma albumin concentrations with glycaemia, insulin sensitivity or insulin secretion (all *p* ≥ 0.1). While there was a positive association of plasma albumin with total cholesterol (*p* = 0.002), no significant relationships with triglycerides, LDL‐ or HDL‐cholesterol were observed (all *p* ≥ 0.3).

### 3.2 Changes During Lifestyle Intervention

**FIGURE 2:**
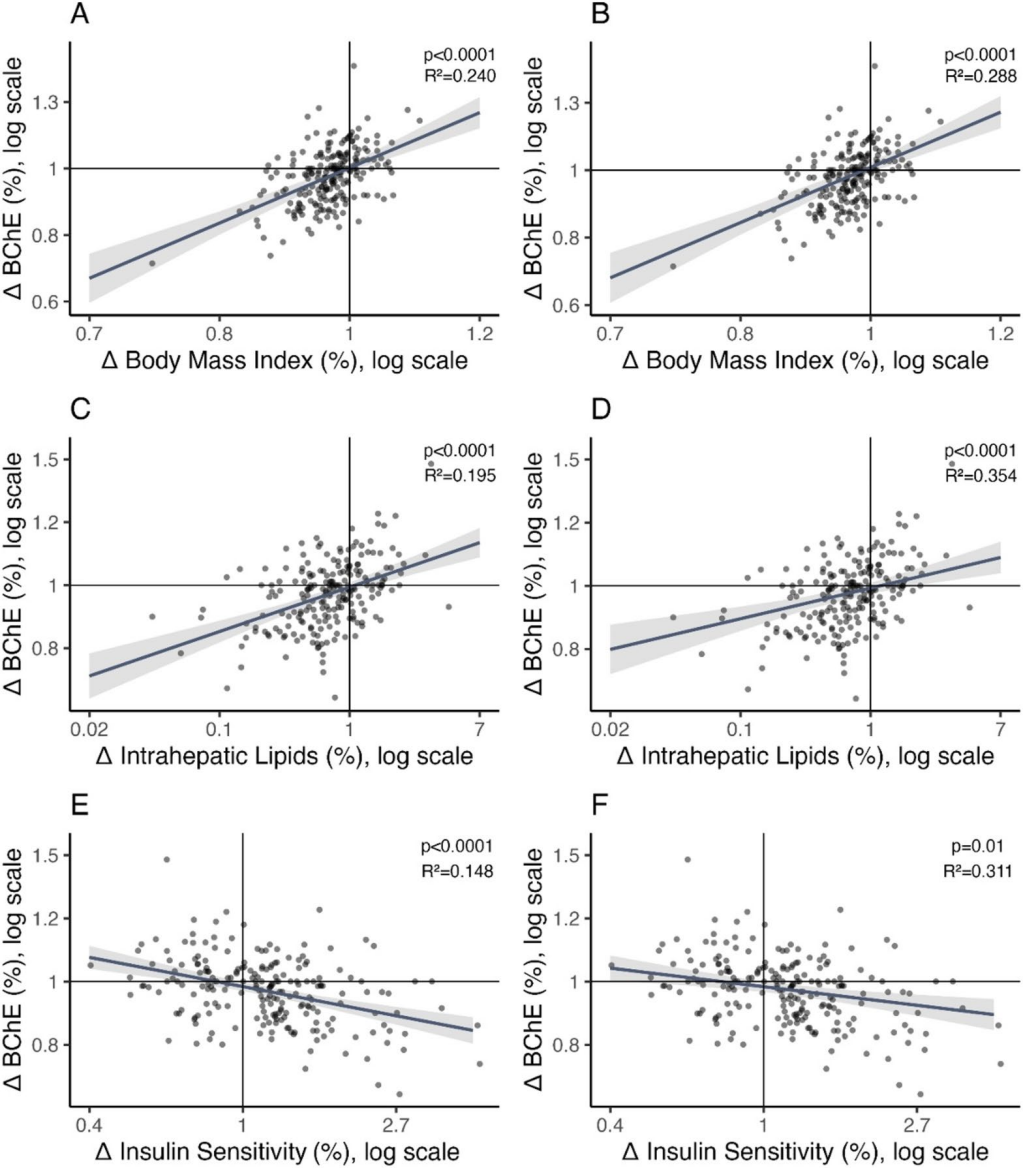
Associations of changes in butyrylcholinesterase activity with changes in BMI, liver fat content and insulin sensitivity during the TULIP lifestyle intervention. Changes from before to after 9 months of lifestyle intervention were calculated. The reduction in BMI was associated with a reduction in butyrylcholinesterase (BChE) (A), which remained significant after adjustment for sex, age, as well as preintervention BMI, BChE and liver fat content (B). Reduction in liver fat content was correlated with a reduction in BChE (C), even after adjustment for sex, age, change in BMI as well as preintervention BMI, BChE and liver fat content (D). Improvement of insulin sensitivity was linked to a reduction in BChE (E). This remained significant after adjustments for sex, age, change in BMI as well as preintervention BMI, BChE, insulin sensitivity (F). Plotted are marginal effects from multivariable regression models, including regression lines and 95% CI (*N* = 215). Data were log‐transformed prior to statistical analyses. Axes indicate fold‐changes of nontransformed data, where a value of 1 indicates no change, values > 1 indicate increases and values < 1 indicate decreases. *p* values and *R* ^2^ are from (multivariable) linear regression analyses. Δ—change during the 9‐month lifestyle intervention, BChE—butyrylcholinesterase, BMI—body mass index, CI—confidence interval.

BChE activity was measured in 215 subjects before and after the 9‐month TULIP lifestyle intervention that combined exercise and diet modification [13]. Most of these participants achieved a weight reduction (*N* = 167, 77.7%) that was associated with a reduction in BChE activity (*p* < 0.0001, Figure 2A). This association remained significant after adjustment for sex, age, as well as preintervention BMI, BChE activity and liver fat content (*p* < 0.0001, Figure 2B).

**FIGURE 3:**
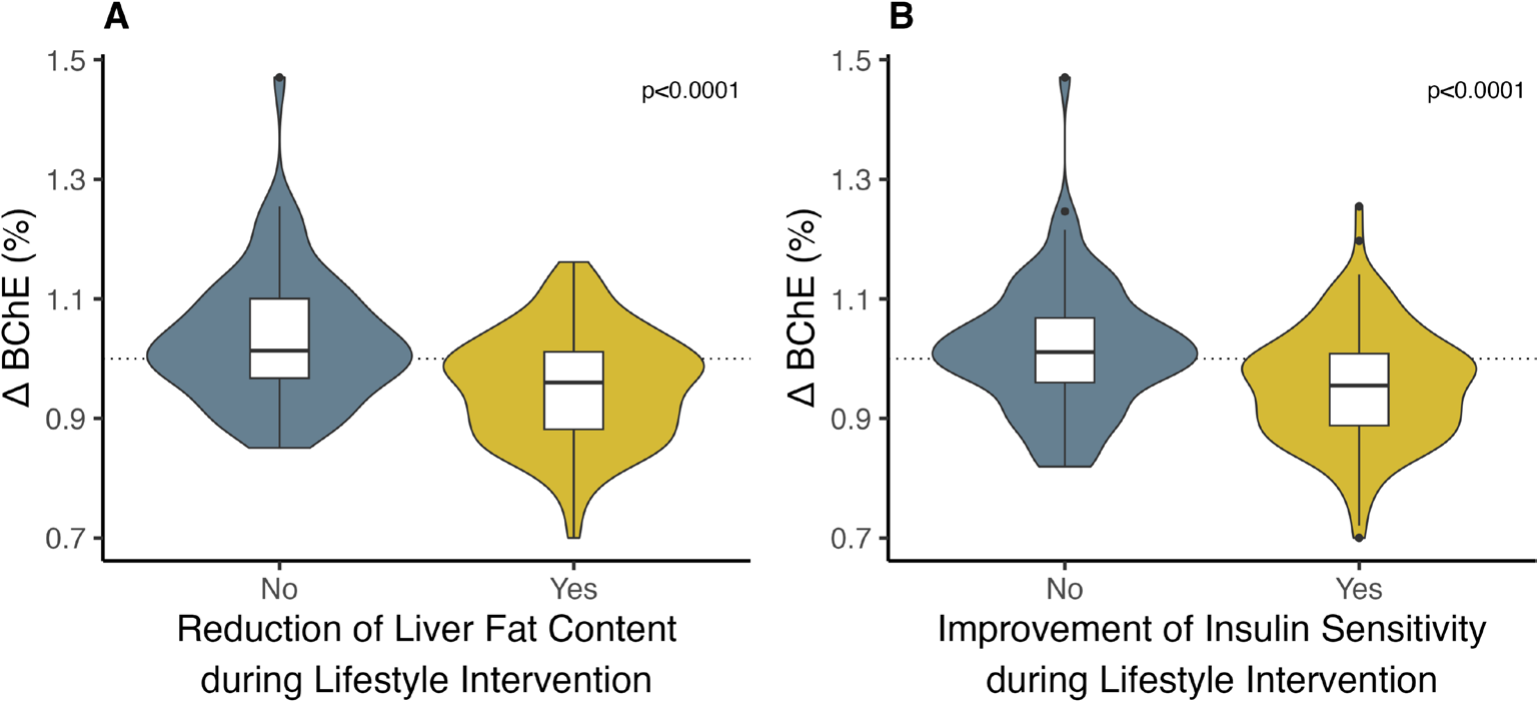
Butyrylcholinesterase activity and response to lifestyle intervention—results from the TULIP study. Persons who achieved a reduction of liver fat content during 9 months of lifestyle intervention, defined as any decrease in liver fat content (A) and/or improved their insulin sensitivity, defined as any increase in the Matsuda Index (B) had a stronger reduction of BChE during the program. Data are presented as box‐violin plots with whiskers indicating 1.5 times the interquartile range. *p* values are from two‐sided unpaired t‐tests. *N* = 64 (no reduction of liver fat), *N* = 151 (reduction of liver fat), *N* = 77 (no improvement of insulin sensitivity), *N* = 138 (improvement of insulin sensitivity). BChE—butyrylcholinesterase.

During lifestyle intervention, most of the participants also achieved a reduction of liver fat content (*N* = 151, 70.2%), that was associated with a decrease in BChE (*p* < 0.0001, Figure 2C), independent of sex, age, the change in BMI as well as preintervention liver fat, BChE and BMI (*p* < 0.0001, Figure 2D). The association between the change of liver fat content and the change of BChE activity was independent of the change in insulin sensitivity (*p* < 0.0001). Hence, those who achieved a reduction of liver fat content had a stronger reduction of BChE activity (*p* < 0.0001, −0.3 kU/L [IQR 1.1] vs. 0.1 kU/L [IQR 1.0], Figure 3A).

Improvement of insulin sensitivity during lifestyle intervention was also associated with a reduction of BChE (*p* < 0.0001, Figure 2E), independent of sex, age, the change in BMI as well as preintervention insulin sensitivity, BChE and BMI (*p* = 0.001, Figure 2F). The association between change in insulin sensitivity and change in BChE was independent of the change in liver fat content (*p* < 0.0001). Those who achieved improvements in insulin sensitivity had a stronger reduction in BChE (*p* < 0.0001, Figure 3B).

For replication of these longitudinal data, we quantified BChE activity in 116 participants of the independent 6‐month lifestyle intervention NUPREDM study that applied diet modification [14]. Here, comparable associations were observed between changes in BChE and improvements in BMI (*p* < 0.0001), liver fat content (*p* = 0.02) and insulin sensitivity (*p* = 0.002), respectively.

### 3.3 Liver Fat Content and *BCHE* Expression in Human Liver Samples

Triglyceride content in human liver samples was positively associated with *BCHE* mRNA expression, the gene encoding the circulating BChE (*p* = 0.007, Figure S2).

## 4 Discussion

Our study confirms that higher BChE activity is associated with liver fat accumulation and is present in patients with the metabolic syndrome. While previous smaller studies had already suggested such relationships [3, 4, 6, 7], our present study provides further clarity on the relationship between BChE activity and glycaemia. We detected relationships between BChE activity and both glucose tolerance and insulin sensitivity, which were independent of liver fat content. In addition to these cross‐sectional findings, we detected a close relationship between the reduction in BChE and metabolic improvements in two independent lifestyle intervention studies.

To quantify liver fat content in vivo, we used ^1^H‐MRS that allows a precise quantification over a wide range of lipid content [18]. Plasma BChE activity was robustly and positively correlated with liver fat content, even in the normal range. In human liver biopsies, *BCHE* mRNA expression was associated with liver fat content, suggesting a transcriptional upregulation during liver fat accumulation. Of note, the relationship between BChE and liver fat content was independent of overall adiposity. Thus, elevated BChE levels in lean individuals may indicate fatty liver, which is less frequent [19] and, therefore, often overlooked in lean persons. This can be of great relevance, as these lean people with fatty livers appear to be at particularly high risk of cardiovascular death [20]. Thus, high BChE levels could prompt further testing for fatty liver in this population. However, the underlying mechanism for the positive relationship between BChE and liver fat content in our study and other studies remains unclear, as genetic knockout of BChE resulted not in less but in more hepatic fat, at least under a high‐fat diet [21]. Although BChE is not a simple indicator of liver fat, it may also reflect liver inflammation. Future studies should explore this potential link to better understand its role in liver disease progression.

Fatty liver is closely linked to insulin resistance. An interesting finding from our study is that BChE activity is related to both liver fat content and insulin resistance, but these associations appear to be independent of each other. This finding suggests that BChE activity may be a useful estimate for both elevated liver fat content and insulin resistance, regardless of the presence of the other factor. These data, furthermore, indicate that BChE could be involved in metabolic processes in hepatocytes that are crucial for glucose metabolism, independent of hepatic steatosis. These processes most likely do not represent liver synthesis capacity per se, as no comparable associations were detected for albumin, another well‐established readout of liver synthesis. Furthermore, recent studies have explored the role of BChE as a predictive marker for systemic complications after surgery. For instance, Verras and Mulita demonstrated that lower postoperative BChE levels are associated with an increased risk for surgical site infections following colorectal surgery [22, 23].

The detected link between BChE and insulin resistance in our study is consistent with the results of smaller studies involving patients with and without overt diabetes, where BChE activity was found to be negatively associated with insulin sensitivity [3, 6, 8]. It is likely that the link between BChE and insulin resistance underlies the associations we observed between higher BChE levels and worse glucose metabolism. For the other major determinant of glycaemia, insulin secretion, we detected no independent associations. Thus, our data do not support previous speculations about the positive effects of BChE on pancreatic beta cells [7]. In line with our findings on glucose metabolism, higher BChE activity was reported to be linked to an increased risk for future type 2 diabetes [24].

Of notice, genetic modulation of BChE in animals indicates a causal role of BChE in regulating systemic insulin sensitivity and glucose tolerance through processes that involve the central nervous system [25]. While these genetic manipulations with absent and restored BChE are not directly transferable to our observations with elevated BChE, there is accumulating evidence that the human central nervous system contributes to systemic glucose metabolism and liver fat [26]. However, as BChE does most likely not cross the blood–brain barrier [27], the relevance of central nervous actions for our current findings remains unclear. Undoubtedly, more mechanistic studies are needed to uncover the mechanisms that underlie the detected links between BChE, liver fat content and systemic glucose metabolism.

Although the great potential of lifestyle interventions to improve human health is very clear, it is also well established that not everyone benefits equally [28]. While the achieved weight loss is easily measurable, other health benefits are often more difficult to detect and require time‐consuming, burdensome and expensive measurements. The beneficial effects of lifestyle interventions are not necessarily and always related to weight loss [13, 29, 30]. Therefore, other readily determinable measures of such benefits would be an important advance towards personalised lifestyle interventions. Our current longitudinal findings from two large lifestyle interventions and observations from a small study with a hypocaloric diet [4] indicate that BChE activity could have such potential. Measuring BChE might be one way to monitor improvements in insulin sensitivity and liver fat content through lifestyle intervention, from simple blood samples.

Among the limitations of our current work is its correlative nature that can never establish causality. Furthermore, we did not include patients with liver diseases (other than MASLD) and can, therefore, not exclude other relationships in such patient groups.

In conclusion, our cross‐sectional and longitudinal results confirm that higher BChE activity is an estimate of liver fat accumulation and that it is present in patients with the metabolic syndrome. Furthermore, we detected links of BChE activity to glucose tolerance and insulin sensitivity, independent of liver fat content. This suggests that BChE could not just be a marker for liver steatosis, but may indicate processes in hepatocytes that contribute to impaired glucose metabolism. Further investigations are needed to clarify the mechanistic contribution and potential diagnostic value of elevated BChE activity in hepatic steatosis, insulin resistance and related metabolic diseases.

## Author Contributions

M.H., J.H., L.R. and A.P. researched and analysed data; J.M. and F.S. researched and analysed MRI data; L.F. and R.W. researched data and contributed to the discussion; E.S. and A.L.B. contributed to the discussion; A.F., H.‐U.H. and N.S. contributed to the design of the trials and discussed data. M.H. and A.P. designed the study, supervised the project and drafted the manuscript together with J.H. All authors contributed to the discussion and approved the final version of the manuscript prior to submission.

## Ethics Statement

The analyses presented in this manuscript are not part of prespecified primary or secondary analyses from any clinical study. Instead, they represent secondary and exploratory analyses conducted using data obtained during the course of previous studies. These studies have been previously published and are appropriately referenced in the methods section of the manuscript. As the original ethical approvals pertain solely to the primary objectives of those initial studies, we have not included the original ethical applications and approvals in this submission.

## Conflicts of Interest

Outside of the current work, R.W. reports lecture fees from Novo Nordisk and Sanofi. R.W. served on an advisory board for Akcea Therapeutics, Daiichi Sankyo, Sanofi and Novo Nordisk. Outside of the current work, M.H. reports research grants from Boehringer Ingelheim and Sanofi to the University Hospital of Tübingen, participation in an advisory board for Boehringer Ingelheim and Sanofi, and lecture fees from Amryt, Bayer, Sanofi, Eli Lilly, Novo Nordisk and Boehringer Ingelheim. The other authors have nothing to disclose.

## Supporting information


Data S1.

## Data Availability

The data generated during the current study are not publicly available because they contain information that could compromise research participant privacy and consent. This paper does not report original code.
