# Supplementary material for: Elevated Cholinesterase Activity and the Metabolic Syndrome—Dissecting Fatty Liver, Insulin Resistance and Dysglycaemia
Source: Liver Int. 2025 Apr 17;45(5):e70046. doi: 10.1111/liv.70046 (PMC12005068; doi:10.1111/liv.70046)
Supplement: Supplementary file 1 — Data S1. [file LIV-45-0-s001.docx]

| **TULIP Cohort NUPREDM Cohort**  N=215 N=116 | | | | | |
| --- | --- | --- | --- | --- | --- |
|  | Median/N | IQR |  | Median/N | IQR |
| Sex  Female  Male | 128  87 |  |  | 75  41 |  |
| Age (years) | 47 | (39, 54) |  | 42 | (32, 50) |
| BMI (kg/m²) | 29.0 | (26.5, 31.9) |  | 30.2 | (28.4, 32.5) |
| Intrahepatic fat, MRS-derived (%) | 3.1 | (1.4, 7.0) |  | 3.5* | (1.8, 11.0) |
| Matsuda insulin sensitivity index (OGTT-derived) | 15.4 | (9.5, 22.7) |  | 13.2 | (8.1, 17.8) |
| Butyrylcholinesterase (kU/l) | 8.4 | (7.3, 10.1) |  | 9.0 | (7.7, 10.8) |

**Elevated cholinesterase activity and the metabolic syndrome - dissecting fatty liver, insulin resistance and dysglycemia**

**Supplementary Material**

**Table S1**: Participant Characteristics - Lifestyle Intervention Cohorts

*n=103. BMI: body mass index; MRS: magnetic resonance spectroscopy; OGTT: oral glucose tolerance test.

| N=238 | Median/N | IQR |
| --- | --- | --- |
| Sex  Female  Male | 91  147 |  |
| Age (years) | 65 | (58, 71) |
| BMI (kg/m²) | 24.0 | (21.2, 26.6) |
| Triacylglycerol (mg)/ 100 mg tissue (%) | 1.6 | (0.9, 3.1) |

**Table S2:** Patient Characteristics – Patients undergoing liver surgery

BMI: body mass index; MRS: magnetic resonance spectroscopy.

| Human Gene | Upstream Primer | Downstream Primer |
| --- | --- | --- |
| *RPS13* | 5′-CCCCACTTGGTTGAAGTTGA-3′ | 5′-ACACCATGTGAATCTCTCAGGA-3′ |
| *BCHE* | 5′- TCCCGTCGTCTGTGATACTG-3′ | 5′- ACTCGTAGCATGGTCTGCTG-3′ |

**Table S3:** List of primers used for PCR and real-time PCR

*BCHE*: butyrylcholinesterase mRNA; PCR: polymerase chain reaction; *RPS13*: 40S ribosomal protein S13 mRNA.

**Table S4:** Associations of butyrylcholinesterase activity with parameters of
glucose and lipid metabolism

|  | Std. ß | Std. error | p _(unadj.)_ | p _(adj. sex, age, BMI)_ | p _(adj. sex, age, BMI, liverfat)_ |
| --- | --- | --- | --- | --- | --- |
| Fasting glucose (mmol/l) | 0.331 | 0.033 | <0.0001 | <0.0001 | <0.0001 |
| 2-h glucose (mmol/l) | 0.251 | 0.033 | <0.0001 | <0.0001 | <0.0001 |
| Area under the glucose curve, 0-120 min (mmol/l) | 0.318 | 0.033 | <0.0001 | <0.0001 | <0.0001 |
| Matsuda insulin sensitivity index (OGTT-derived) | -0.371 | 0.032 | <0.0001 | <0.0001 | <0.0001 |
| AUC C-Peptide_(0-30 min)_/ AUC Glucose_(0-30 min)_ | 0.154 | 0.034 | <0.0001 | 0.7* | 0.5* |
| Triglycerides (mg/dl) | 0.373 | 0.032 | <0.0001 | <0.0001 | <0.0001 |
| Cholesterol (mg/dl) | 0.254 | 0.033 | <0.0001 | <0.0001 | <0.0001 |
| LDL-Cholesterol (mg/dl) | 0.254 | 0.033 | <0.0001 | <0.0001 | <0.0001 |
| HDL-Cholesterol (mg/dl) | -0.259 | 0.033 | <0.0001 | <0.0001 | <0.0001 |

*Additionally adjusted for Matsuda insulin sensitivity index. Data were scaled prior to analyses. P-values and standardized ß are from multivariable linear regression models. BMI: body mass index; OGTT: oral glucose tolerance test; AUC: area under the curve; LDL-Cholesterol: low-density lipoprotein cholesterol; HDL-Cholesterol: high-density lipoprotein cholesterol.


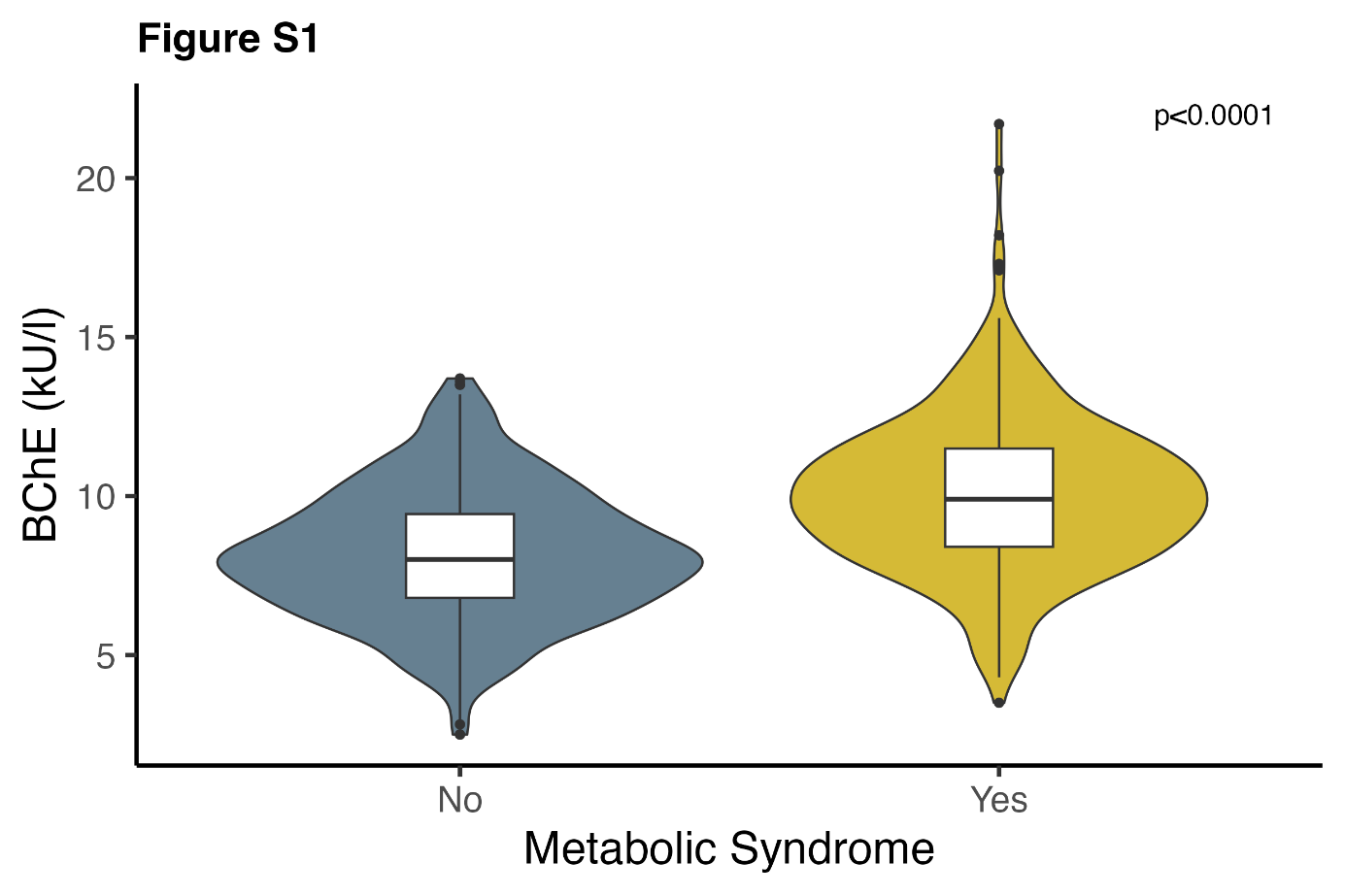


Figure S1: Butyrylcholinesterase activity and the metabolic syndrome

BChE activity was higher in persons with the metabolic syndrome. Data are presented as box-violin plots with whiskers indicating 1.5 interquartile range. Data was log transformed prior to statistical analyses. P values are from two-sided unpaired t-tests. N=496 (no metabolic syndrome), N=343 (metabolic syndrome). BChE – butyrylcholinesterase.


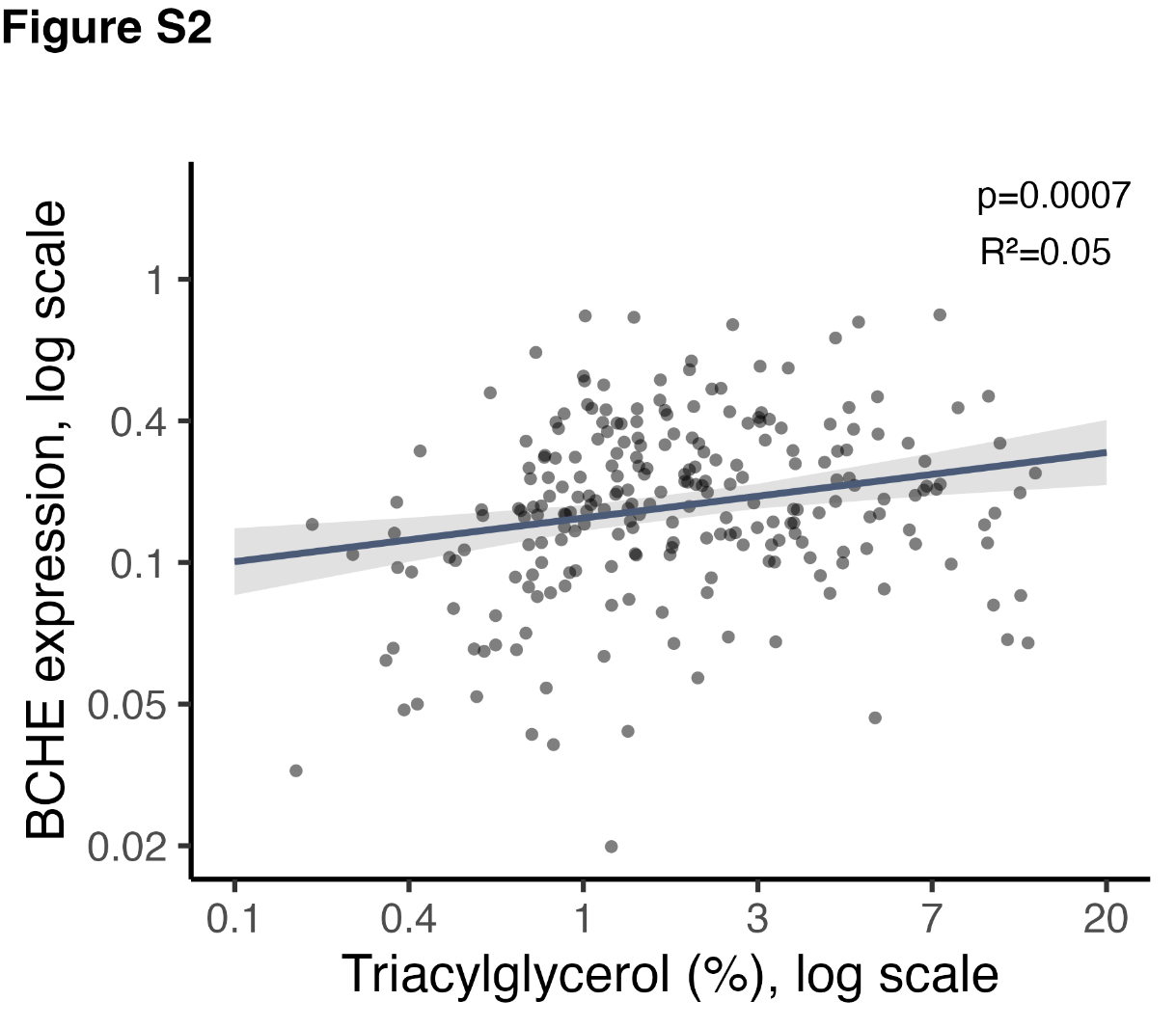


Figure S2: Association of *BCHE* mRNA expression with triacylglycerol content in human liver samples

*BCHE* expression was positively associated with triacylglycerol content in human liver samples.

Plotted are individual data points, the regression line and 95% CI (N=238). *BCHE* mRNA expression was normalized to *RPS13*. Data was log transformed prior to statistical analyses. P values and R² are from a linear regression analysis. *BCHE* - butyrylcholinesterase mRNA, CI – confidence interval, *RPS13* - 40S ribosomal protein S13 mRNA.
